# Endovascular management of pancreatitis-related pseudoaneurysms: A review of techniques

**DOI:** 10.1371/journal.pone.0191998

**Published:** 2018-01-29

**Authors:** Bartosz Zabicki, Nattakarn Limphaibool, Marte Johanne Veilemand Holstad, Robert Juszkat

**Affiliations:** Department of Diagnostic and Interventional Radiology, Poznan University of Medical Sciences, Poznan, Poland; Medical University Innsbruck, AUSTRIA

## Abstract

**Objectives:**

To present the various techniques used in the management of pancreatitis-related pseudoaneurysms of visceral vessels.

**Methods:**

The retrospective clinical study was carried out at the Department of Diagnostic and Interventional Radiology at Poznan University of Medical Sciences from 2011 to 2016. The fifteen patients included in the study were diagnosed with pseudoaneurysms of visceral arteries, as a complication of chronic pancreatitis. The diagnosis was made using contrast-enhanced computed tomography, followed by angiography. On admission, all patients were symptomatic, with varying degrees of abdominal pain. One patient was haemodynamically unstable. Treatments with endovascular techniques were analysed, along with their efficacy and outcomes. Coil embolisation was performed in 5 patients. Stent graft was used in 1 patient. Liquid embolic agents were used in 7 cases, of which 5 patients were treated with thrombin injection and 2 with Squid. A combination of techniques was used in 2 patients.

**Results:**

The most common artery affected by pseudoaneurysm formation was the splenic artery (7/15; 46.7%), and the size of the pseudoaneurysms ranged from 27 mm to 85 mm. Primary technical success was achieved in 14 out of 15 patients (93.3%). One patient required reintervention. Two patients required splenectomy after embolisation due to splenic ischemia. No recanalisation was present at the follow-up computed tomography performed after 1 to 3 weeks, and no mortality was observed within 30 days.

**Conclusion:**

Vascular complications of pancreatitis require accurate diagnosis and immediate treatment. Endovascular intervention is highly effective and is the preferred treatment option. The technique used is determined based on vascular anatomy and the patient’s haemodynamic status.

## Introduction

Following pancreatic inflammation, the spread of proteolytic fluids, causing weakening of local vessel walls, may lead to the formation of arterial pseudoaneurysms. The formation of pseudoaneurysms occurs with a higher incidence in chronic pancreatitis (4% - 8%) than in acute pancreatitis (accurate prevalence not clearly defined in recent literature) [1–3]. Pseudoaneurysms most commonly affect the splenic artery, followed by gastroduodenal, pancreaticoduodenal, gastric, and hepatic arteries [4]. Regardless of their size, pseudoaneurysms present with a significant risk of spontaneous rupture and fatal haemorrhages, accompanied by a mortality rate reported to approach 100% [5, 6]. Immediate diagnosis and definitive treatment is therefore necessary, irrespective of the size of the pseudoaneurysm and the patient’s symptoms. This study presents and analyses the different endovascular techniques used in the management of 15 patients with pseudoaneurysms arising as a complication of chronic pancreatitis.

## Materials and methods

### Patients

The retrospective clinical study was carried out in the Department of Diagnostic and Interventional Radiology at Poznan University of Medical Sciences from 2011 to 2016. We analysed 15 patients, aged 29–69 years, who were diagnosed with visceral artery pseudoaneurysms resulting from underlying chronic pancreatitis. The diagnosis of chronic pancreatitis was established based on the clinical cardinal symptoms and supporting radiological findings [7]. The diagnosis was made in the gastroenterology department before referral to our department.

A total of 15 patients with visceral artery pseudoaneurysms presented at our institution for treatment after referral from the gastroenterology department in the five-year time frame. Inclusion criteria for patients included in this study were clinical and radiological diagnosis of underlying chronic pancreatitis (computed tomography (CT)) and evidence of pseudoaneurysm formation in visceral artery (CT angiography).

Patient characteristics (medical record number, age, gender) were recorded in a database and retrospectively collected and made anonymous by one member of the interventional radiology team before analysis.

Fourteen patients were haemodynamically stable and presenting with abdominal pain, of varying degrees of severity. One patient was admitted with symptoms of hypovolemic shock (systolic blood pressure below 60 mmHg) and massive haematemesis, due to the splenic artery rupturing into the stomach as a result of prior gastrocystostomy. The choice of endovascular therapeutic intervention was made based on the size and anatomical location of the pseudoaneurysm. Table 1 lists the affected vessels, pseudoaneurysm diameter, and embolic agent(s) used.

**Table 1:** Arterial site of involvement, size of pseudoaneurysms, and treatment administered.

Case	Arterial site	Size	Treatment
1	Splenic artery	34 mm	Squid embolisation
2	Right gastroepiploic artery	71 mm	Thrombin embolisation
3	Superior Mesenteric Artery	78 mm	Stent graft (Bentley)
4	Splenic Artery	34 mm	Coil embolisation
5	Coeliac artery trifurcation	27 mm	Coil embolisation
6	Splenic Artery	33 mm	1. Squid embolisation 2. Splenectomy
7	Right hepatic Artery	35 mm	Thrombin embolisation
8	Gastroduodenal Artery	37 mm	Coil embolisation
9	Common hepatic artery	61 mm	Thrombin and coil embolisation
10	Common hepatic artery variant	60 mm	1. Coil embolisation 2. Vascular plug (St. Jude Medical)
11	Splenic artery	42 mm	Thrombin embolisation
12	Right gastroepiploic artery	45 mm	Thrombin embolisation
13	Splenic artery	85 mm	1. Thrombin and coil embolisation 2. Splenectomy
14	Splenic artery	48 mm	Coil embolisation
15	Splenic artery	51 mm	Thrombin embolisation

### Ethics

In accordance with Polish law and GCP regulations, this retrospective case series did not require approval of the Bioethics Committee at Poznan University of Medical Sciences, and is not defined as a medical experiment. All data were fully anonymized before being analysed by researchers, and therefore separate informed consent to use them in an anonymized form was not required for the purpose of this report.

### Diagnostic imaging and interventional techniques

The diagnosis of a pseuodoaneurysm was made by contrast-enhanced CT in all 15 patients. Subsequent use of angiography confirmed the diagnosis and located the arterial source of the pseudoaneurysm. Angiographic catheters (Simmons and Cobra) served as guiding catheters for microcatheters (Vasco 10 Balt, Rebar 14 Covidien) in the endovascular intervention. Access was gained through the common femoral artery in 13 procedures, and brachial artery access was used in 1 case of a superior mesenteric artery (SMA) pseudoaneurysm. Both access sites were used in 1 case, with initial coil embolisation via the femoral artery followed by vascular plug insertion via the brachial artery. Follow-up CT examinations were performed on the following day and again 1–3 weeks after the procedures.

Detachable microcoils (Concerto®, Covidien, Irvine, CA, USA) were delivered via microcatheter in cases in which the pseudoaneurysm neck was accessible. This was carried out using the sandwich technique or by direct delivery into the pseudoaneurysm sac. The sandwich technique was utilised in the pseudoaneurysms of the splenic arteries (34 mm, 48 mm) and the gastroduodenal artery (37 mm), due to their expendable nature. Direct delivery of coils into the sac was required in the case of a pseudoaneurysm of the coeliac artery trifurcation (27 mm) and was the initial technique utilised in the pseudoaneurysm of the common hepatic artery variant (60 mm).

Stent grafts provided an exclusion of the pseudoaneurysm in situations in which the parent artery was nonexpendable, such as the SMA. The 6-mm balloon-expandable stent graft (Bentley InnoMed GmbH, Hechingen, Germany) was utilised to keep the vessel patent. It was delivered through a 6F 90cm sheath (Destination, Terumo) via the brachial artery access.

Bovine thrombin (Biotrombina, Biomed, Lublin, Poland) was injected through a microcatheter into the pseudoaneurysm. No percutaneous injections with ultrasound (US) guidance were carried out. In cases in which arterial patency was required and a collateral supply was not yet established, thrombin was used, alone or in conjunction with coils, to occlude the pseudoaneurysm cavity. Thrombin injection was used in 5 patients presenting with pseudoaneurysms in various coeliac branches, including the gastroepipoloic, right hepatic, splenic, and common hepatic artery. The amount of thrombin used was determined based on pseudoaneurysm size and the size of its perfusion zone. In pseudoaneurysms with a perfusion zone diameter of up to 20 mm, 1 ampoule of thrombin (2 ml of 200U/ml per ampoule) was initially utilized. Pseudoaneurysms with perfusion zones above 20 mm were initiated with 2 ampoules of thrombin. Thrombin injection was adjusted according to the neck size and flow velocity in each particular case. Additional ampoules were added, when necessary, to stabilize the ongoing thrombosis. An extra diagnostic run was performed 20 minutes after the procedure to exclude immediate revascularisation.

In cases in which the pseudoaneurysm neck was not easily accessible or the patient’s own coagulation was insufficient, a non-adhesive liquid embolic agent (SquidPeri 18, Emboflu, Fribourg, Switzerland) was used. Standard viscosity SquidPeri 18 formula was injected proximal to the pseudoaneurysm neck, using a DMSO-compatible microcatheter, to effectively block any feeding vessels distal to the injection site. Embolisation was continued until occlusion of affected vessel segment was achieved.

Amplatzer vascular plug (AVP) type II (Amplatzer®, St Jude Medical, Plymouth, MN, UK) was delivered through the JR4.0 6 F guide catheter (Launcher, Medtronic, United States) advanced through a 6F sheath access site in the left brachial artery. Two AVPs II were used in one case of a pseudoaneurysm of the common hepatic artery. The vascular plugs were deployed proximal and distal to the pseudoaneurysm neck, causing the self-expanding Nitinol meshes to completely seal off the pseudoaneurysm from the feeding vessel. As the high-velocity blood flow of the hepatic artery increases the risk of non-target embolisation when using liquid embolic agents and coils, the AVPs were deemed a more optimal choice. The plugs provide a well-controlled and stable occlusion, and is a more cost-effective option when compared to the utilization of a large number of coils and stent grafts in this scenario. AVPs are also appropriate for the common hepatic artery due to its flexible delivery system when compared to stent grafts.

## Results

Diagnostic CT identified the splenic artery as the most common pseudoaneurysm site (7/15; 46.7%) in this study, followed by the common hepatic artery (2/15; 13.3%), and right gastroepiploic artery (2/15; 13.3%). Measurements of pseudoaneurysm diameter ranged from 27 mm to 85 mm, with an average of 57.3 mm.

**Fig 1:**
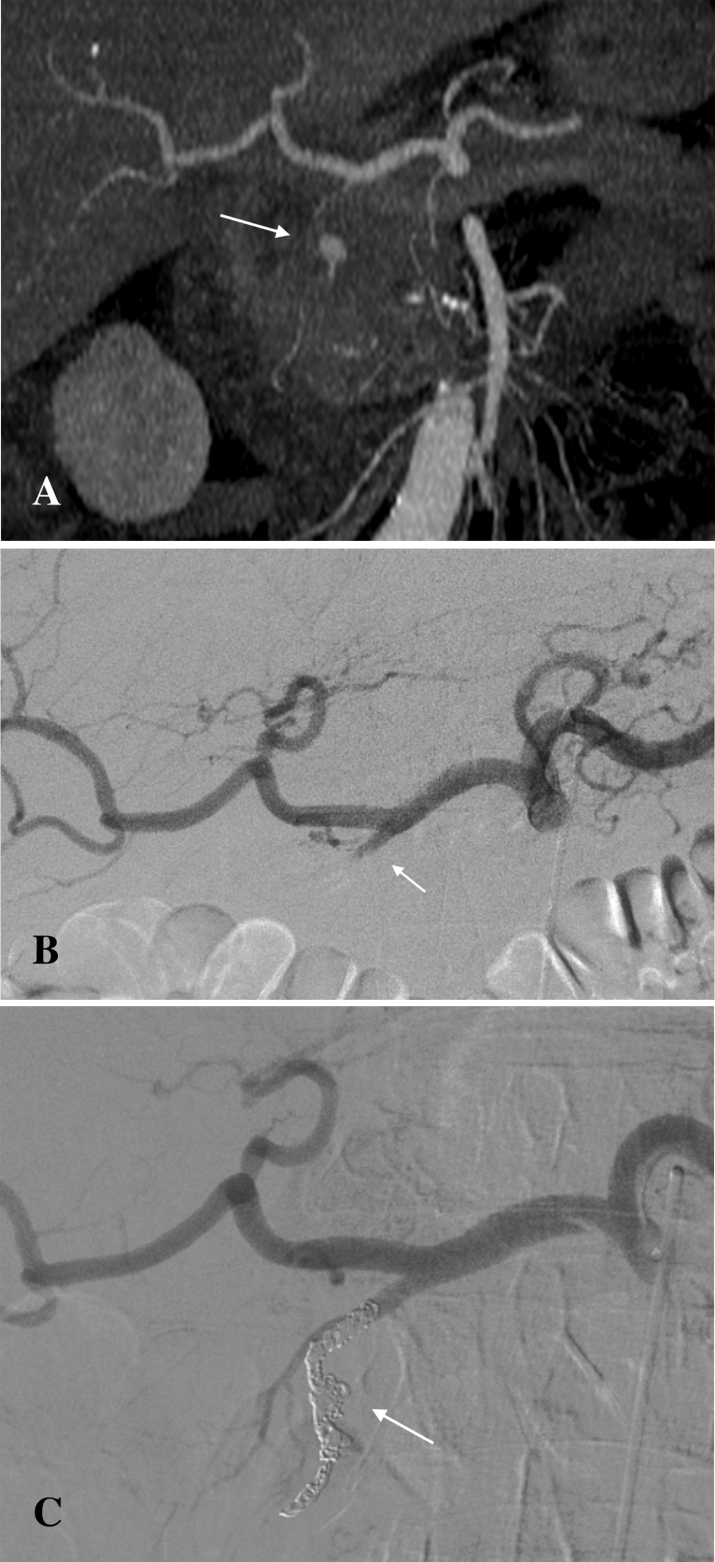
Pseudoaneurysm of the gastroduodenal artery pre- and post-coil embolization. (A) Pseudoaneurysm of the gastroduodenal artery visible on the contrast-enhanced CT (arrow). (B) Digital Subtraction Angiography (DSA) image showing vasospasm and thrombosis of the gastroduodenal artery with no active bleeding (arrow). (C) Post-embolisation DSA image showing successful coil embolisation of the gastroduodenal artery (arrow).

Catheter-directed endovascular delivery of microcoils was utilised in 5 cases. In 2 cases of a pseudoaneurysm of the splenic artery, the sandwich method was successfully employed. This technique was effective in preventing backflow from the collateral circulation. No ischemic complications were observed, as collateral circulation to the spleen was sufficient. The sandwich technique was also utilised in a case of a gastroduodenal artery pseudoaneurysm. The pseudoaneurysm showed thrombosis and a bleeding area of 10 mm, with vasospasm of the originating artery. Saline injection was initially used to expose the bleeding site before coil delivery (Fig 1A, 1B and 1C). A pseudoaneurysm of the coeliac artery trifurcation was treated with coil embolisation of the pseudoaneurysm sac. This technique maintained the patency of the parent vessel and its branches.

**Fig 2:**
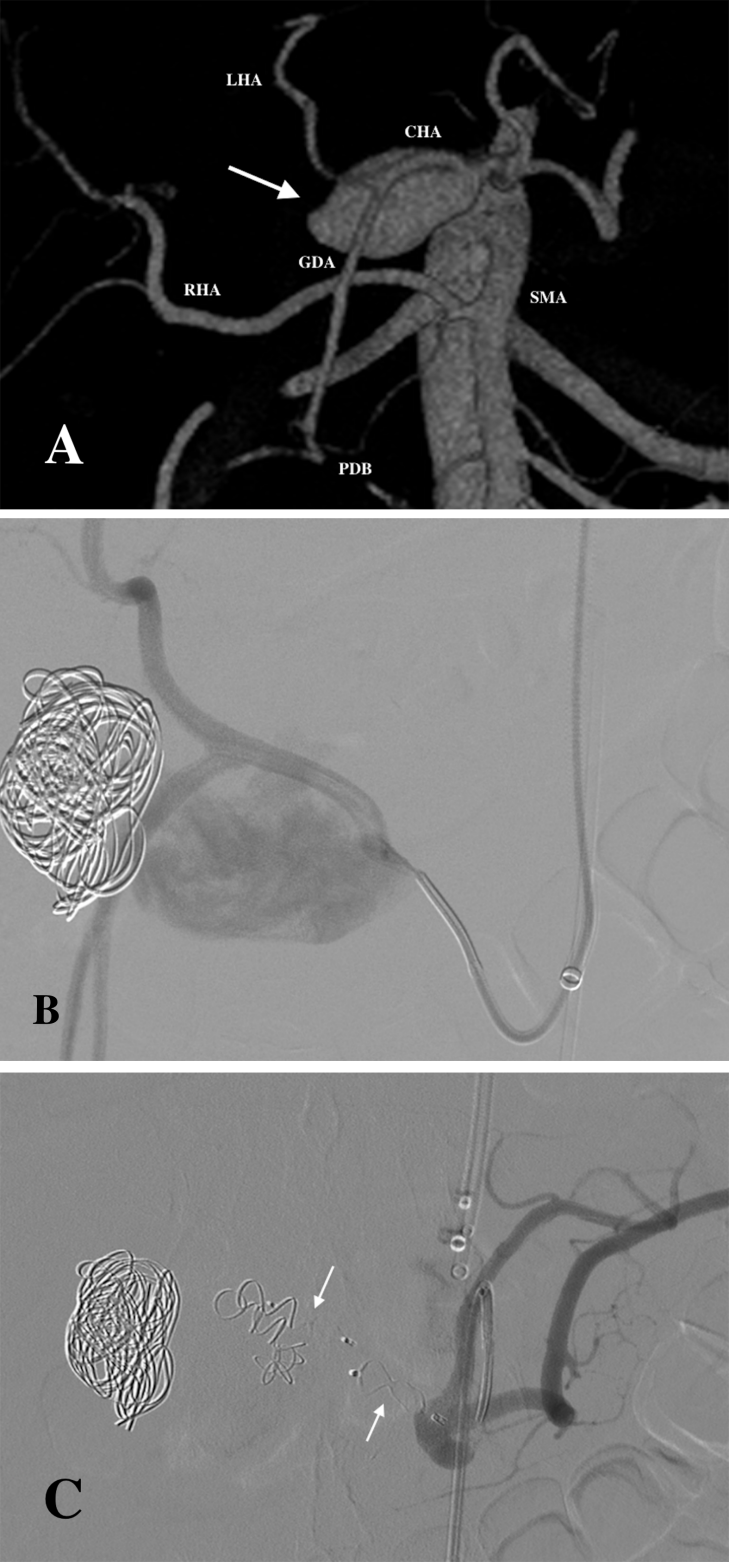
Pseudoaneurysm of the common hepatic artery variant treated initially with coil embolisation followed by vascular plugs placement. (A) 3D Volume Rendering CT image showing a pseudoaneurysm (arrow) arising from the common hepatic artery (CHA). This anatomical variant shows the right hepatic artery (RHA) arising from the superior mesenteric artery (SMA). The common hepatic artery branches into the gastroduodenal artery (GDA) and left hepatic artery (LHA). Patent pancreaticoduodenal branches (PDB) are present connecting the SMA and the GDA. (B) DSA image showing ineffective coil embolisation of the pseudoaneurysm. (C) Final DSA depicting procedural success after the placement of two vascular plugs.

In the case of a pseudoaneurysm involving the common hepatic artery (60 mm), the initial method of intervention was coil embolisation. This proved to be ineffective when enlargement of the pseudoaneurysm was later observed. The patient had an anatomical variant, in which the right hepatic artery originated from the SMA (Fig 2A and 2B). This anatomical variant enabled the use of two AVP II to occlude the common hepatic artery, effectively treating the pseudoaneurysm with no resulting ischemia to the liver (Fig 2C).

**Fig 3:**
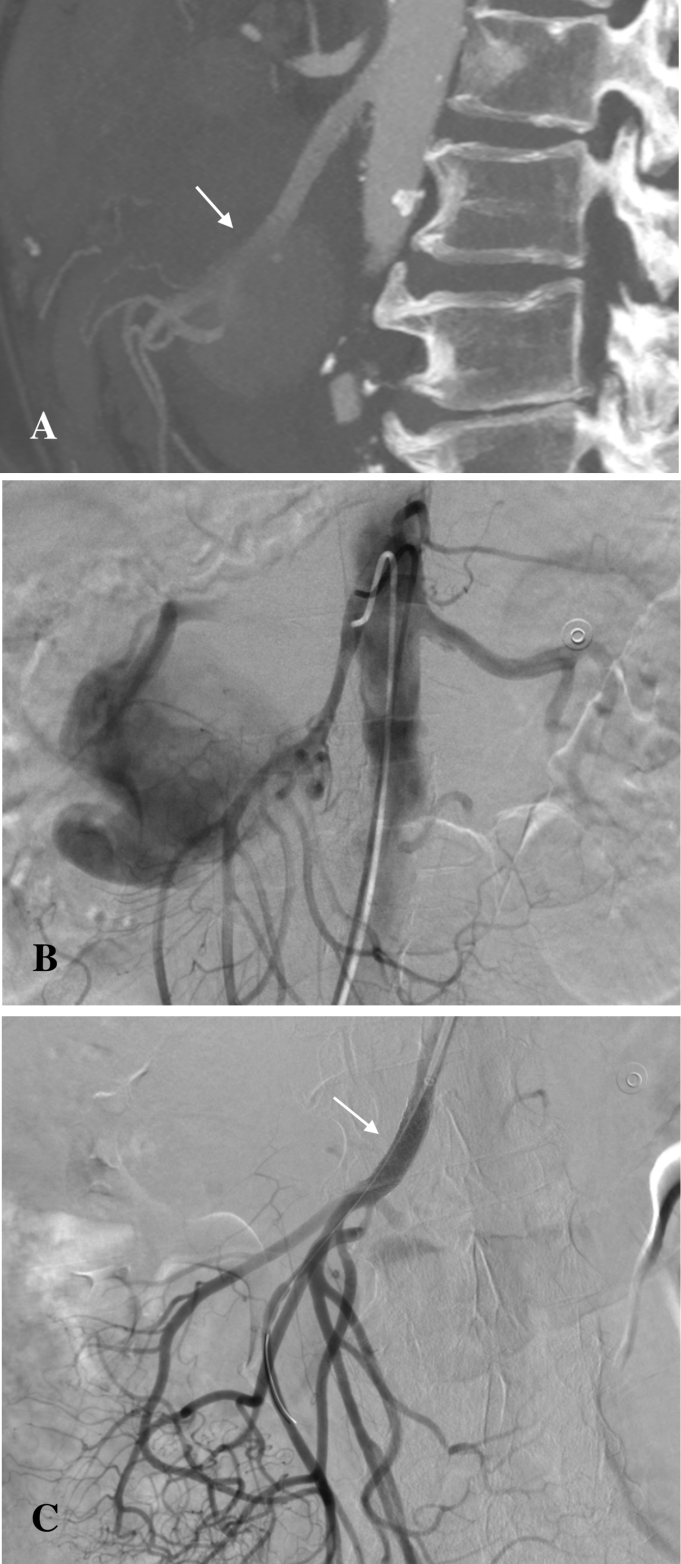
Superior mesenteric artery pseudoaneurysm treated with stent graft implantation. (A) Contrast-enhanced CT scan showing the pseudoaneurysm arising from the SMA (arrow). (B) DSA showing pseudoaneurysm. (C) Final selective DSA confirming the exclusion of the pseudoaneurysm following stent graft implantation and post-dilation.

Stent graft was used in the treatment of a pseudoaneurysm of the SMA (Fig 3A and 3B). Persistent bleeding at SMA trunk was observed due to discrete leakage after initial implantation. The stent graft was subsequently post-dilated, and extravasation was no longer visible (Fig 3C). Stent graft expansion sacrificed the right colic artery. Adequate perfusion of gastrointestinal tissue was maintained through the vessel’s extensive collateral circulation.

**Fig 4:**
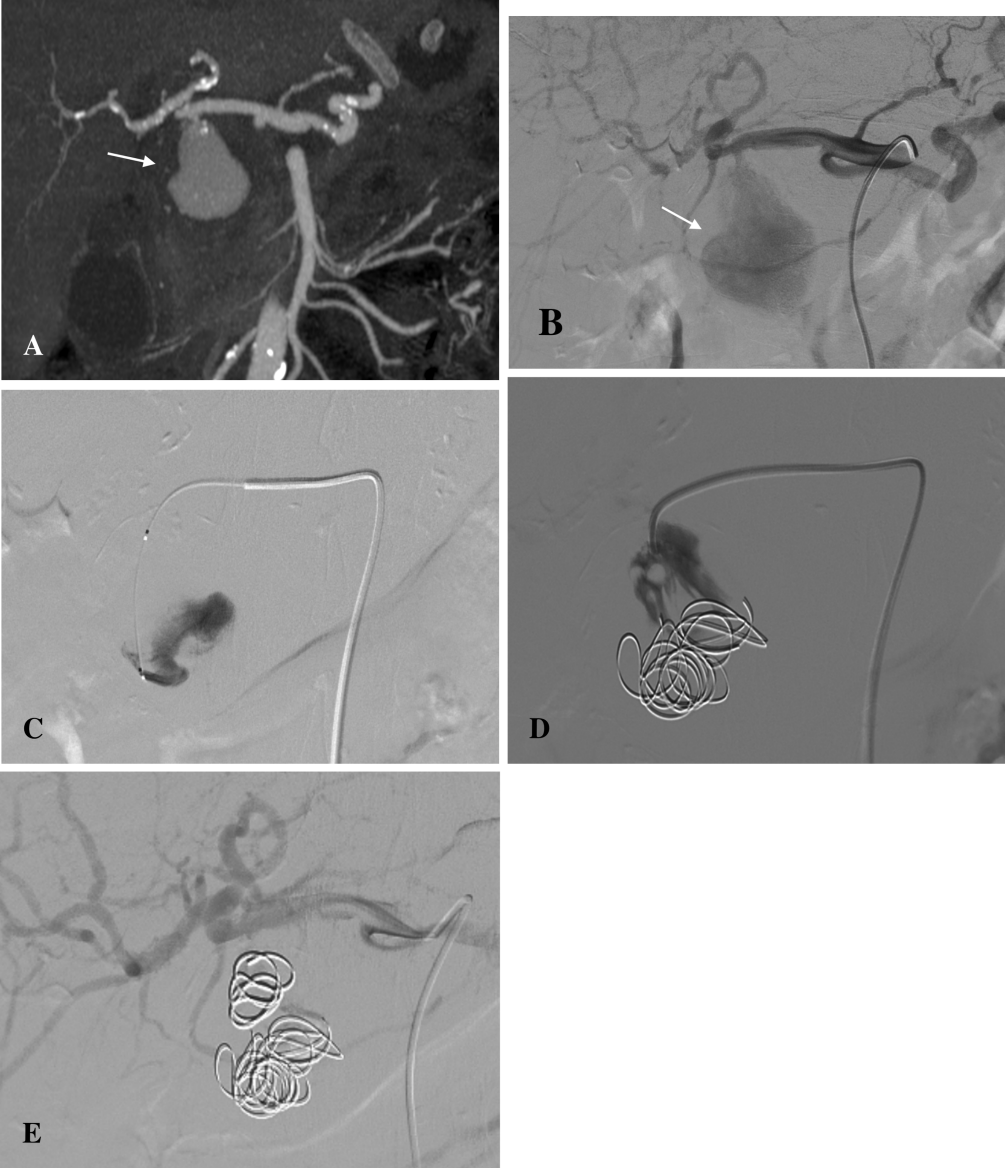
Pseudoaneursym of the common hepatic artery treated with coil and thrombin embolization. (A) Contrast-enhanced CT scan and (B) DSA image showing the pseudoaneursym arising from the distal common hepatic artery (arrow). (C) DSA image showing initial thrombin injection. (D) Persistent perfusion despite additional placement of coils. (E) Final DSA depicting procedural success after the deployment of an additional coil and thrombin.

Bovine thrombin injections were administered alone in 5 cases. Two additional cases combined coils and thrombin to stabilise the thrombus. In the case of a pseudoaneurysm of the distal common hepatic artery, thrombin injection directly into the pseudoaneurysm was unsuccessful due to high blood flow within the sac. Coils were therefore administered, reducing the velocity of the blood flow within the sac and facilitating thrombosis (Fig 4A, 4B, 4C, 4D and 4E). In a case requiring urgent endovascular management, thrombin was used in conjunction with coils. The patient presented with haematemesis and unstable vital signs, with a systolic blood pressure of 60 mmHg after a prior gastrocystostomy. The splenic artery was bleeding into the pseudocyst, and from there bleeding into stomach was observed. Two ampoules of thrombin were initially administered to the pseudoaneurysm sac, together with coil deployment over the neck. When the pseudoaneurysm started showing signs of recanalisation, 2 additional ampoules of thrombin were administered to the splenic artery as an attempted bailout procedure to stop bleeding. A follow-up CT, performed the following day, showed splenic infarction with total occlusion of the splenic artery along with and its distal collateral supply. Clinical symptoms of left epigastric pain were present in addition to the radiological findings. Splenectomy was therefore indicated.

**Fig 5:**
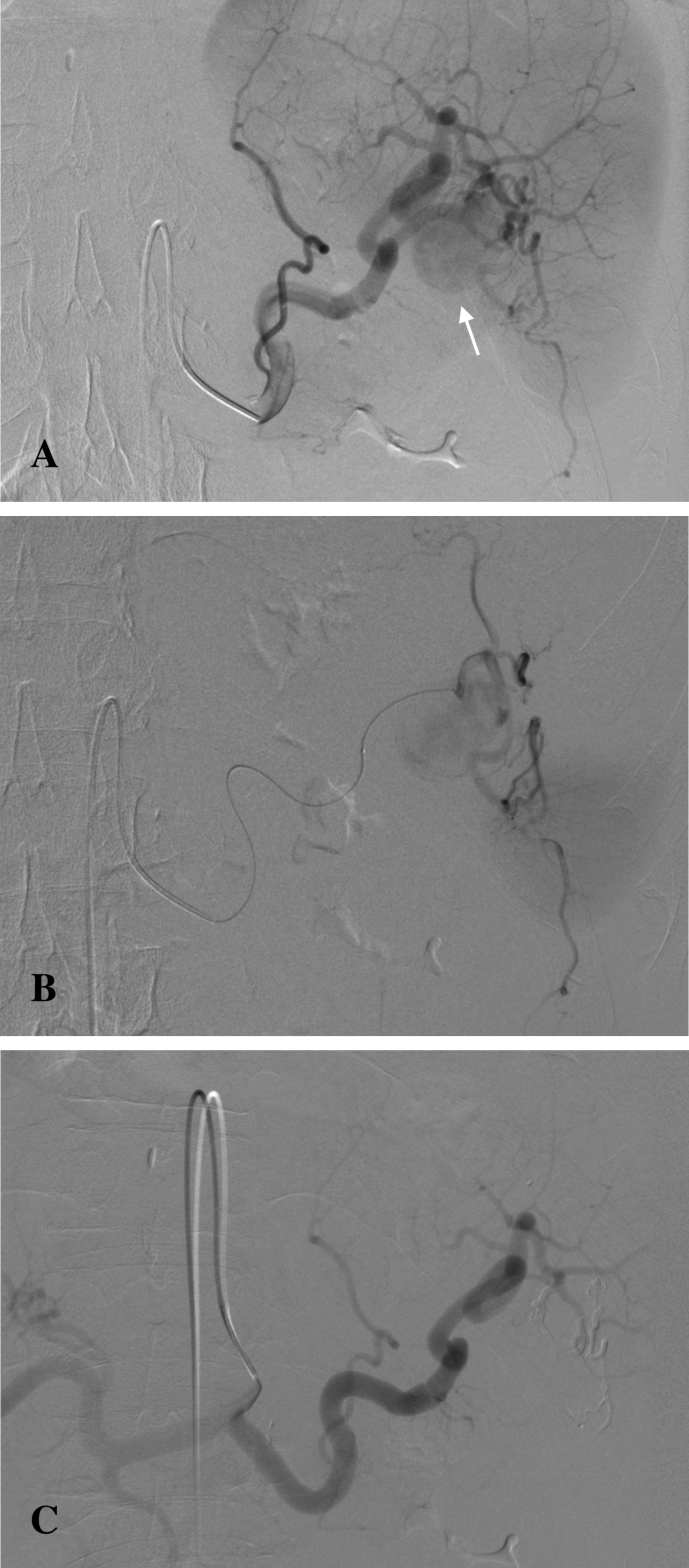
Splenic artery pseudoaneursym treated with squid injection. (A) DSA image showing the pseudoaneurysm arising from the distal splenic artery near the splenic hilum (arrow). (B) Microcatheter position before liquid embolic agent (Squid) injection to the distal splenic artery. (C) DSA image post-embolisation showing the Squid cast and an absence of contrast extravasation.

Squid embolisation was used in 2 patients. The first case was a pseudoaneurysm involving the splenic artery (34 mm), near the hilum, and was treated successfully using the liquid non-adhesive agent (Fig 5A, 5B and 5C). The second case was a pseudoaneurysm of the distal splenic artery (33 mm). Total blockage of splenic arterial perfusion was observed as a result of insufficient control over injected agent. Splenectomy was indicated and performed on the same day due to symptomatic, significant splenic infarction.

Primary success, defined by complete exclusion of pseudoaneurysm from systemic circulation after initial intervention, was achieved in 14 out of 15 cases (93.3%). Reintervention was required in 1 case, where a successful outcome was observed after secondary treatment. Complications were observed in 2 cases, in which splenectomy was indicated.

Follow-up imaging was done using contrast-enhanced CT on the following day and within 3 weeks of the procedure. Abdominal CT showed no evidence of recanalisation or bleeding in any of the treated patients in the post-interventional follow-up period. No further intervention besides two splenectomies was necessary. There was no periprocedural mortality, defined by death occurring within 1 month of procedure. None of the patients were referred back to our hospital after the follow-up period due to recurrence of pseudoaneurysm.

## Discussion

Endovascular procedures allow rapid control of bleeding through minimally invasive techniques. It is a common initial choice in the treatment of pancreatitis-related pseudoaneurysms, with a reported success rate ranging from 79–100% [2, 3, 8, 9]. This was clinically consistent with the outcomes presented in this study, in which final technical success was achieved in all cases, regardless of the patient’s haemodynamic status.

Preference for a specific technique takes into consideration the anatomical location of the affected vessel, the size of pseudoaneurysm sac, and the patient's haemodynamic status. Contrast-enhanced CT is a highly sensitive imaging technique in the primary detection of pseudonauerysm extravasation. Subsequent angiography allowed the evaluation of the anatomy of the visceral vessels and their collateral circulation, and measurement of the pseudoaneurysms prior to choosing the optimal endovascular technique. Several studies have shown that angiography is extremely valuable in locating bleeding pseudoaneurysms, with sensitivity rates of over 90% [10, 11]

Treatment with coil embolisation can be administered with minimal risk of gastrointestinal ischemia in expendable arteries. These include arteries with an extensive collateral circulation, such as the splenic and gastroduodenal arteries. The sandwich technique employed in three cases successfully excluded the pseudoaneurysms in all cases. A clinical success rate of approximately 89% has been reported in current literature [12]. Detachable coils used in the procedure provided better control and more precise deployment than the pushable coils, and could be retrieved if inappropriately placed [13]. The requirement of normal coagulation status for successful embolisation also meant that coiling was not effective in cases of hypovolemic shock or underlying coagulopathy [14].

Nonexpendable arteries required the preservation of distal blood flow. Embolisation of the pseudoaneurysm sac itself was applicable in cases of a narrow neck, such as in the pseudoaneurysm of the coeliac artery trifurcation. In cases of a wide neck, placing coils directly into the sac posed a significant risk of coil migration into the parent artery, reported in other literature to occur in up to 3% of cases [9]. In these cases, stent graft placement was utilised.

The use of stent graft in the treatment of a SMA pseudoaneurysm maintained the patency of the nonexpendable artery, preventing bowel ischemia. This technique has a long-term patency rate of approximately 82% [15]. Brachial artery access was utilised in this procedure as it facilitated cannulation of SMA through a less convoluted route. Large proximal SMA segments are favourable in the utilisation of the stent graft as its placement requires a stiffer delivery and cannot pass through small tortuous vessels. Covered stents are preferable in arteries with a diameter of at least 6 mm, as smaller vessels are associated with a higher risk of thrombosis [16]. The larger diameter of the SMA is therefore an advantage. Stent thrombosis occurs more frequently in patients with underlying chronic diseases, including diabetes mellitus, chronic kidney disease, acute coronary disease, and in patients who are current smokers [17]. Other potential complications include loss of vessel branches after stent placement [13].

Thrombin injection is another technique used particularly for pseudoaneurysms that are not easily accessible using a transcatheter technique, and in which the parent vessel cannot be identified [18]. If diagnostic imaging is unable to identify the parent vessel, thrombin can be injected percutaneously directly into the lumen of the pseudoaneurysm using US guidance. Thrombosis may be ineffective if blood flow within the lumen is high. If the neck is accessible, coils can then be inserted into the pseudoaneurysm cavity to slow down blood flow and facilitate thrombin-induced thrombosis [6]. This combination of techniques was particularly useful in a case of the large common hepatic artery pseudoaneursym (61 mm), where patency of the parent vessel was crucial. Efficacy is also dependent on the patient’s own coagulation [5]. Non-adhesive liquid embolic agents (such as Squid) are preferred in cases in which coagulation is inadequate, but thrombin is still favourable as the initial treatment in critical conditions until other techniques can be prepared. If the patient is haemodynamically unstable, another treatment option should be considered, as thrombin may not properly embolise the parent vessel or pseudoaneurysm sac. Complications arising from the use of thrombin as an embolic agent include increased risk of arterial thrombosis, arteriovenous fistulas, and history of allergic reactions to bovine thrombin [5, 6, 13]. No allergic reactions to thrombin were observed in this study. Many instances of recanalisation have been reported in current literature, highlighting the importance of follow-up in these patients [19, 20, 21].

Liquid non-adhesive ethylene vinyl alcohol (EVOH) copolymers (e.g. Squid and Onyx) provide a high degree of control when treating pseudoaneurysms, but require the operator to be familiar with the solution used. It is highly effective in occluding vessels distal to the injection site, especially when anatomy does not allow for direct access to pseudoaneurysm sac. EVOH copolymers are not dependent on the patient’s coagulation status, unlike thrombin and coils, and can be used to embolise a vessel even if the patient’s coagulation is disrupted. The primary disadvantage of this treatment option is the difficulty associated with administration and the possibility of human error, especially in critical situations. If the injection is not well-controlled, non-target embolisation may lead to ischemic complications, which was observed in our case of a pseudoaneurysm of the splenic artery treated with Squid embolic agent. Risk of such complications is even higher when using adhesive embolic agents such as n-butyl-cyanoacrylate embolic glue (NBCA). An increased risk is also seen when the target vessel has a high blood flow, making the injection harder to control [6].

The use of AVPs is ideal for the embolisation of large vessels with high flow. In these vessels, limited control of coils or a liquid embolic agent can lead to coil migration and non-target embolisation, respectively. The larger delivery systems of AVPs and their limited flexibility make them problematic for smaller and more convoluted vessels, an exception being AVP 4, the size and flexibility of which enables it to be delivered to more distal and tortuous vasculature. The manufacturer’s recommendation to oversize the plugs by 30–50% relative to the size of target vessel significantly decreases the risk of device migration [9, 22]. The possibility of recapturing and repositioning the AVP after deployment provide more control during the endovascular intervention, as opposed to other embolic agents currently used. The vascular plugs have a shorter occlusion time when compared with coils, and have been reported to contribute to the decrease in total procedure time by an average of 13.3 minutes [9, 23, 24]. A vessel that would require several coils can be embolised using a single AVP, contributing to a significant reduction in procedure costs [9, 25]. Studies reported a 30–71% reduction in material costs per patient in the use of AVP II compared to platinum coils [24, 26]. Incidence of efficient embolisation induced by vascular plug is reported to approach 100% in literature, which corresponded to the outcomes of our study [24, 26]. As vascular plugs completely occlude corresponding vessels, it cannot be used in cases of a pseudoaneurysm with a nonexpendable feeding artery. A thorough understanding of the vascular anatomy is therefore crucial to avoid ischemic complications. This study is limited by its small sample size and retrospective nature. Furthermore, only one patient was treated with AVP. Therefore, firm conclusions on the outcomes of this technique cannot be made.

## Conclusion

Pseudoaneurysms of visceral vessels as a complication of chronic pancreatitis are associated with a high mortality risk, requiring urgent intervention which can be achieved through minimally invasive endovascular techniques with a high rate of success. When planning the endovascular procedure, it is essential to take into consideration vascular anatomy, characteristics of the pseudoaneurysm, and the patient’s general condition. In expendable vessels, the sandwich technique with parent vessel occlusion provides an effective and stable occlusion. Embolisation of the pseudoaneurysm sac itself with either coils or liquid embolic agents, as well as complete exclusion using stent grafts, can be considered when patency of the target vessel is desired. It is important to thoroughly assess the target vessel, as high blood flow increases the risk of non-target embolisation, especially if the clinician is unfamiliar with the embolic agent used. The management techniques presented in this study aim to help guide vascular interventionalists in providing the highest standard of care in the treatment of visceral pseudoaneurysms in different clinical scenarios.

## Supporting information

S1 Table Patient database detailing characteristics of pseudoaneurysms. (XLSX)
